# Glass Ionomer Cement Modified by Resin with Incorporation of Nanohydroxyapatite: In Vitro Evaluation of Physical-Biological Properties

**DOI:** 10.3390/nano10071412

**Published:** 2020-07-19

**Authors:** Luis Eduardo Genaro, Giovana Anovazzi, Josimeri Hebling, Angela Cristina Cilense Zuanon

**Affiliations:** Department of Morphology, Genetics, Orthodontic and Pediatric Dentistry, Araraquara School of Dentistry, São Paulo State University (UNESP), Araraquara 14801-903, São Paulo, Brazil; giovanaanovazzi@hotmail.com (G.A.); josimeri.hebling@unesp.br (J.H.); cristina.zuanon@unesp.br (A.C.C.Z.)

**Keywords:** scanning electron microscopy, porosity, glass ionomer cement, cytotoxicity

## Abstract

Resin-modified glass ionomer cement (RMGIC) has important properties. However, like other restorative materials, it has limitations such as decreased biocompatibility. The incorporation of nanoparticles (NP) in the RMGIC resulted in improvements in some of its properties. The aim of this study was to evaluate the physical-biological properties of RMGIC with the addition of nanohydroxyapatite (HANP). Material and Methods: Vitremer RMGIC was used, incorporating HANP by amalgamator, vortex and manual techniques, totaling ten experimental groups. The distribution and dispersion of the HANP were evaluated qualitatively by field emission scanning electron microscope (SEM-FEG). The evaluation of image porosity (SEM-FEG) with the help of imageJ. Cell viability 3-(4,5-dimethylthiazol-2yl)-2,5-diphenyl tetrazoline bromide (MTT) and cell morphology analyses were performed on MDPC-23 odontoblastoid cells at 24 and 72 h. Results: It was possible to observe good dispersion and distribution of HANP in the samples in all experimental groups. The incorporation of 5% HANP into the vortex stirred RMGIC resulted in fewer pores. The increase in the concentration of HANP was directly proportional to the decrease in cytotoxicity. Conclusions: It is concluded that the use of a vortex with the incorporation of 5% HANP is the most appropriate mixing technique when considering the smallest number of pores inside the material. A higher concentration of HANP resulted in better cell viability, suggesting that this association is promising for future studies of new restorative materials.

## 1. Introduction

Glass ionomer cement (GIC) is a dental material that contains fluoraluminosilicate glass in its powder composition of calcium, basic silicon oxide, aluminum oxide and calcium, magnesium, and sodium fluoride. The liquid is an aqueous solution of polyacrylic acid, tartaric acid, and itaconic acid, which has biological compatibility [1], adherence to the tooth structure [2], and antibacterial properties [3], among other desirable properties. Even so, its u se as restorative material is still limited due to its brittleness and low compressive strength [4] when compared to other restorative materials. In order to overcome these limitations, the resin-modified GIC (RMGIC) was developed. In addition to the acid–base reaction, RMGICs have a polymerization reaction as they have resin monomers in their composition [5,6]. In most cases, this polymerization is photo-initiated.

Recently, the incorporation of hydroxyapatite nanoparticles (HANP) to RMGIC has shown promising results such as the increase in adhesive strength to dentin [7]. In addition, it has been shown to favor increased enamel remineralization [8]. Nanoparticles show good results when added to restorative materials [9].

The incorporation of HANP to the conventional GIC at 12% concentration showed improvement in microhardness, resistance to compression, and diametrical traction [10]. Concentrations of 2% and 8% also provided improvements in fracture resistance [7]. Thus, when using RMGIC, the addition of HANP could improve its biological properties, since in vitro tests demonstrate that the cytotoxic effects of RMGIC are more evident when compared to conventional GIC [11,12].

Del Angel-Mosqueda et al. [13] (2018) carried out a cytotoxicity study analyzing gingival fibroblasts, where the photopolymerized medium was treated with 100 mg/mL of hydroxyapatite of lyophilized bone for 24 h at room temperature in response to Vitrebond RMGIC and found that there was an increase in cell viability, suggesting that HA plays a protective role, decreasing the cytotoxic effect.

Studies on the association of NPs and GIC show promising results [7,14,15], however, few have performed cytotoxicity tests [16], which are fundamental and precede the application of the material to the oral cavity. It is known that the American Dental Association (ADA) and the American National Standards Institute (ANSI-ISO-10993) recommend preclinical tests such as cytotoxicity assays developed in cell culture, laboratory animal tests, and finally used on humans [17,18].

The effect of adding NPs depends on its proper distribution and dispersion in the restorative material and there is no research related to different techniques for incorporating NP into the GIC, a technical step that certainly influences the properties of this material, so it is important to conduct research with this theme. We observed studies analyzing only GIC encapsulated in the powder/liquid mixing method using mechanical manipulators in different potentials [19]. The properties of the material are also strongly related to its microstructure, which in turn depends on the size and homogeneous distribution of its particles [20], which result in the quality of its mechanical resistance [21].

The analysis of different methods of incorporation of NP into the GIC is innovative and important, since it will contribute to adjust the conditions of homogeneity of NP distribution and dispersion within the material.

According to Moshaverinia et al. [7], the concentration of 2% HANP added to the GIC demonstrated improvements in its physical and mechanical properties. Additionally, the 5% and 10% concentrations are new to be tested. Thus, these concentrations were chosen to be used in the tests proposed in this study. The development of research on this subject may, in the near future, improve the properties of the material, facilitate clinical procedures, and make them more effective and long lasting.

In this way, it is essential to carry out studies that, aside from evaluating the physical, chemical, and mechanical properties of the association of NPs to the RMGIC, also evaluate biological properties, making it safe and effective for clinical application.

The objective of this work was to evaluate the effect of different techniques of the incorporation of HANP in different concentrations to the RMGIC regarding the distribution and dispersion, porosity, and cytotoxicity.

The null hypothesis of this study is that there is no improvement in the physical-biological properties of glass ionomer cement after the addition of HANP.

## 2. Materials and Methods

### 2.1. Preparation of Test Specimens

In the present work, portions of RMGIC powder from Vitremer (3M-ESPE Dental Products, St. Paul, MN, USA). With the portions of HANP (Sigma-Aldrich, ref. 677418-10g, batch MKBW9108V, St. Louis, MO, USA), both were weighed with the aid of an analytical balance (Gehaka Ltda-model BG 440).

The amount of each material was established so that, upon mixing thereof, the RMGIC associated with 2%, 5%, and 10% by weight HANP could be obtained. This material was stored individually in Eppendorf tubes for the preparation of each test specimen. Afterward, the materials were mixed and the mixing techniques are explained below.

Eight samples were used and the established groups are shown in Table 1.

### 2.2. Mixing Techniques

We mixed the powdered materials according to the techniques below:

Amalgamator. The powder (RMGIC + HANP) was inserted into amalgam capsules. These were submitted to the action of the amalgamator (Ultramat-SDI Brasil Industria e Comercio Ltd., Sao Paulo, Brazil) with vibration for six seconds.

Vortex. The Eppendorf-packed powder (RMGIC + HANP) was placed in vortex (Vortex Phoenix, Ref. 12446, Phoenix Ind. and Com. De Equips Scientists Ltd., Araraquara, Sao Paulo, Brazil) for vigorous mixing for one minute.

Manual. The powder (RMGIC + HANP) was manipulated and mixed in a spatula block using a plastic spatula in all directions for one minute.

After obtaining the HANP-associated GIC (2%, 5%, or 10% by weight), a drop of liquid present in the RMGIC kit was added to the powder portion and spatulation was performed as recommended by the manufacturer. This material was inserted into a silicone matrix that was 3 mm high by 6 mm in diameter, with the aid of a Centrix syringe (DFL and Trade S.A. Rio de Janeiro, RJ, Brazil) to obtain the specimens. A polyester strip with a 1 mm thick glass slide and a weight of 100 g for 30 s.

Then, the photoactivation was performed with the aid of the light curing agent Radii Cal (SDI Brasil Industria e Comercio Ltda., Sao Paulo, SP, Brazil) for one minute with an intensity of 1200 mW/cm^2^. These specimens were stored at 100% humidity in an appropriate container.

### 2.3. Distribution and Dispersion of Nanohydroxyapatite

The distribution assessment after 24 h of sample preparation was performed by scanning electron microscopy (SEM-FEG) (SM-300, Topcon, Tokyo, Japan). The samples were fractured with a surgical hammer and chisel, using a channel made in the center to direct the fracture. The analyzed fragments had a dimension of 2 mm by 2 mm, were coated with a gold–palladium alloy under high vacuum and taken to 500 and 20,000 SEM-FEG magnification for imaging. X-ray spectroscopy was also performed by dispersing energy to identify its chemical elements (EDX) (Shimadzu Corporation, Kyoto, Japan).

### 2.4. Porosity

The specimens were fractured using a surgical hammer and chisel from a channel made in its center for fracture targeting. The analyzed fragments had a dimension of 2 mm by 2 mm, were coated with a gold–palladium alloy under high vacuum and taken to the 100-fold magnification SEM-FEG for imaging, which were analyzed using the ImageJ program (Rasband WS, ImageJ; U.S. National Institutes of Health, Bethesda, MD, USA). All the pores present in the analyzed sample were selected and the total pore area was computed, then this data were analyzed by statistical analysis. All the images obtained were subdivided into quadrants, the area of the first quadrant being analyzed, to avoid a tendency in the results. 

### 2.5. Cell Viability Analysis

The MDPC-23 odontoblast cell was used for feasibility analysis. MDPC-23 cells were grown in Dulbecco’s modified Eagle’s medium (DMEM, Gibco, Grand Island, NY, USA) supplemented with 10% heat-inactivated fetal bovine serum (FBS; GIBCO, Grand Island, NY, USA), 100 IU/mL, and 100 mg/mL, respectively, of penicillin and streptomycin and 2 mmol/L of glutamine (GIBCO, Grand Island, NY, USA) in a humidified atmosphere containing 5% CO_2_ at 37 °C. The cells were subcultured every three days until the number of cells needed to perform the study were obtained using a density of 3 × 10^4^ cells/cm^2^.

The specimens were placed individually in pre-cultured MDPC-23 cells and incubated for 24 h and 72 h. After this period, the extracts were aspirated and the cell viability analysis was performed for the samples. The control group was represented by MDPC-23 cells maintained only in DMEM. The experiment was carried out in duplicate. The samples were stored in an oven at 37 °C with relative humidity.

The cells were incubated for 4 h at 37 °C and 5% CO_2_ with the 3-(4,5-dimethylthiazol-2yl)-2,5-diphenyl tetrazoline bromide (MTT) solution (Sigma-Aldrich Corp., St. Louis, MO, USA) diluted in DMEM (1:10). The formazan crystals that formed in viable cells were then dissolved in acidified isopropanol and the absorbance of the resulting solution was read at 570 nm (Synergy H1, BioTek, Winooski, VT, USA). The mean absorbance of the positive control group was considered to be 100% of cell viability, and the percentage values for the experimental groups were calculated based on this parameter [12].

### 2.6. Analysis of Cell Morphology

In order to perform the cell morphology analysis, the cells were seeded on glass slides placed at the bottom of 24-well plates. In each time interval of analysis (*n* = 2), the cells were fixed in 2.5% glutaraldehyde (Sigma Chemical Co.), and then post-fixed in 1% osmium tetroxide (Sigma Chemical Co.), dehydrated in increasing concentrations of alcohol (30, 50, 70, 90, and 100%) and submitted to chemical drying in HMDS (1,1,1,3,3,3-hexamethyldisilazane; Sigma Chemical Co.). Finally, the slides with the cells were fixed on metal stubs, kept in a desiccator for 72 h, sputter-coated with gold, and finally analyzed by scanning electron microscopy (SEM-FEG) (SM-300, Topcon, Tokyo, Japan).

### 2.7. Statistical Analysis

The data were analyzed by analysis of variance (ANOVA) with fixed criteria. The Tukey test was applied to the pore area data, and the Kruskal–Wallis and Mann–Whitney tests to the cell viability data. The distribution of values and the homogeneity of the data were previously tested. A descriptive analysis was carried out for cell distribution, dispersion, and morphology. For all tests, the level of significance was 5%.

## 3. Results

**Figure 1:**
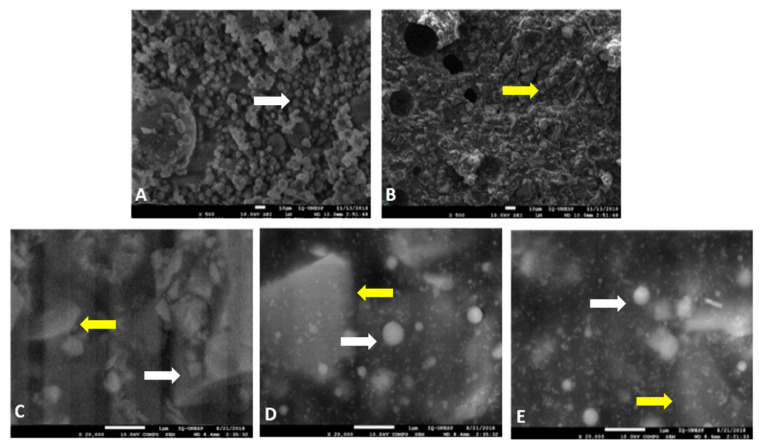
Distribution of nanohydroxyapatite within glass ionomer cement modified by resin. Legend: The white arrows indicate the HANP and the yellow arrows indicate the particles of the RMGIC. (**A**) Microscopic image of spherical HANP structures with magnification × 500. (**B**) Microscopic image in group C with magnification × 500. (**C**) Microscopic image of group A2 with × 20,000 magnification. (**D**) Microscopic image of the V5 group with a magnification of × 20,000. (**E**) Microscopic image of the M10 group with a magnification of × 20,000.

It can be seen that there was a good distribution of HANP within the RMGIC, regardless of the concentration and manipulation technique, as demonstrated in Figure 1.

There was no difference between the distribution of the HANP among the experimental groups. This demonstrates that regardless of the technique for incorporating HANP into the RMGIC, there was good distribution within the material.

**Figure 2:**
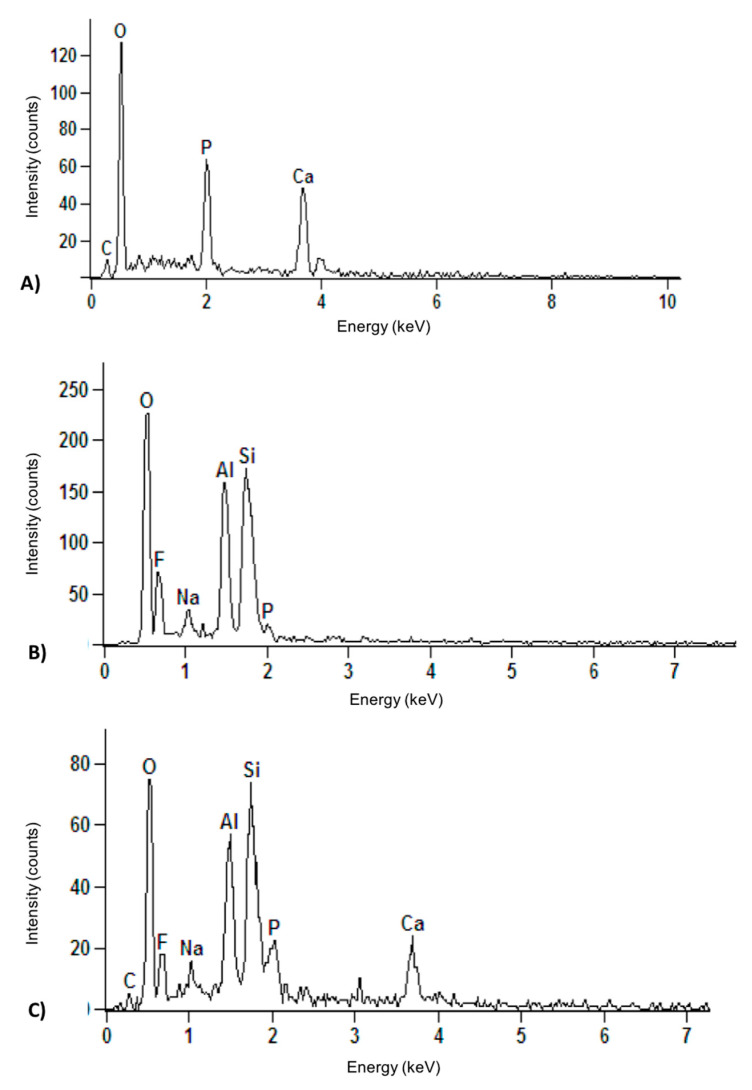
X-ray dispersive energy spectroscopy of the experimental groups. Legend: (**A**) Chemical composition of HANP by means of x-ray dispersive energy spectroscopy. (**B**) Chemical composition of the control group by means of x-ray dispersive energy spectroscopy. (**C**) Chemical composition of groups containing 2%, 5%, and 10% HANP + RMGIC by means of x-ray dispersive energy spectroscopy.

X-ray dispersive energy spectroscopy (Figure 2) demonstrates the chemical components of HANP, RMGIC, and the experimental groups HANP + RMGIC, which had the same spectrum. It can be seen that HANP was rich in Ca and P ions, while RMGIC had a greater amount of Al and Si ions.

**Figure 3:**
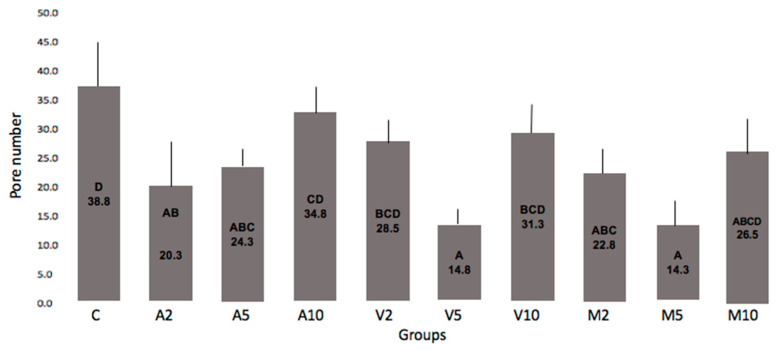
Number of pores of the experimental groups. Legend: Number of pores of the experimental groups. The columns represent averages and the bars represent standard deviations. The columns identified with the same letter do not differ statistically (Tukey, *p* 0.061).

**Figure 4:**
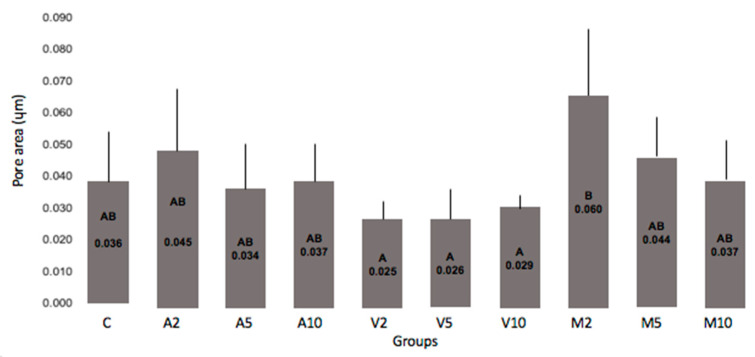
Pore area of experimental groups. Legend: Pore area of experimental groups. The columns represent averages and the bars represent standard deviations. The columns identified with the same letter do not differ statistically (Tukey, *p* 0.068).

**Figure 5:**
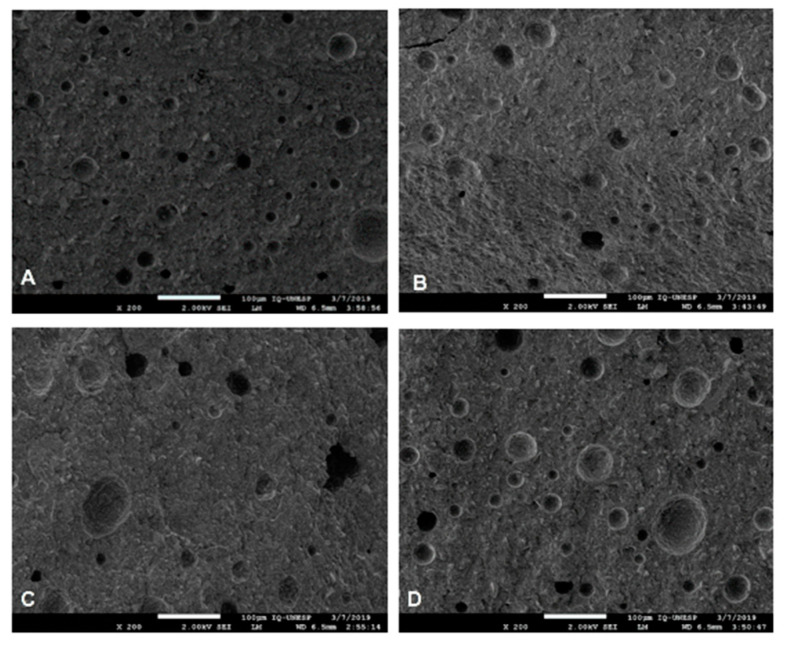
Distribution of the pores of the experimental groups inside the material. Legend: (**A**) Distribution of the pores of the control group. (**B**) Distribution of pores in group V5. (**C**) Distribution of pores in the M5 group. (**D**) Pore distribution in the A10 group.

Data related to the number and area occupied by the pores as a function of the HANP concentration incorporated into the RMGIC powder and the mixing technique are shown in Figure 3, Figure 4 and Figure 5.

It can be seen that all groups incorporated with 10% HANP had a number of pores similar to the control. Meanwhile, a significantly smaller number of pores was found for the other groups compared to the control. Groups V5 or M5 resulted in a smaller number of pores than the number observed in group C. None of the groups differed from C in relation to the area occupied by the pores, however, a smaller value was observed among the groups submitted to the vortex.

**Figure 6:**
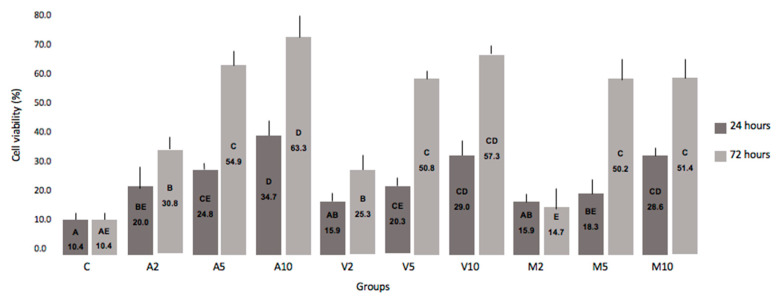
Cellular viability at 24 h and 72 h. Legend: Cell viability after contact of specimens with MDPC-23 cells for 24 and 72 h. No statistical difference between time periods (Kruskal–Wallis and Mann–Whitney tests, *p* 0.67).

**Figure 7:**
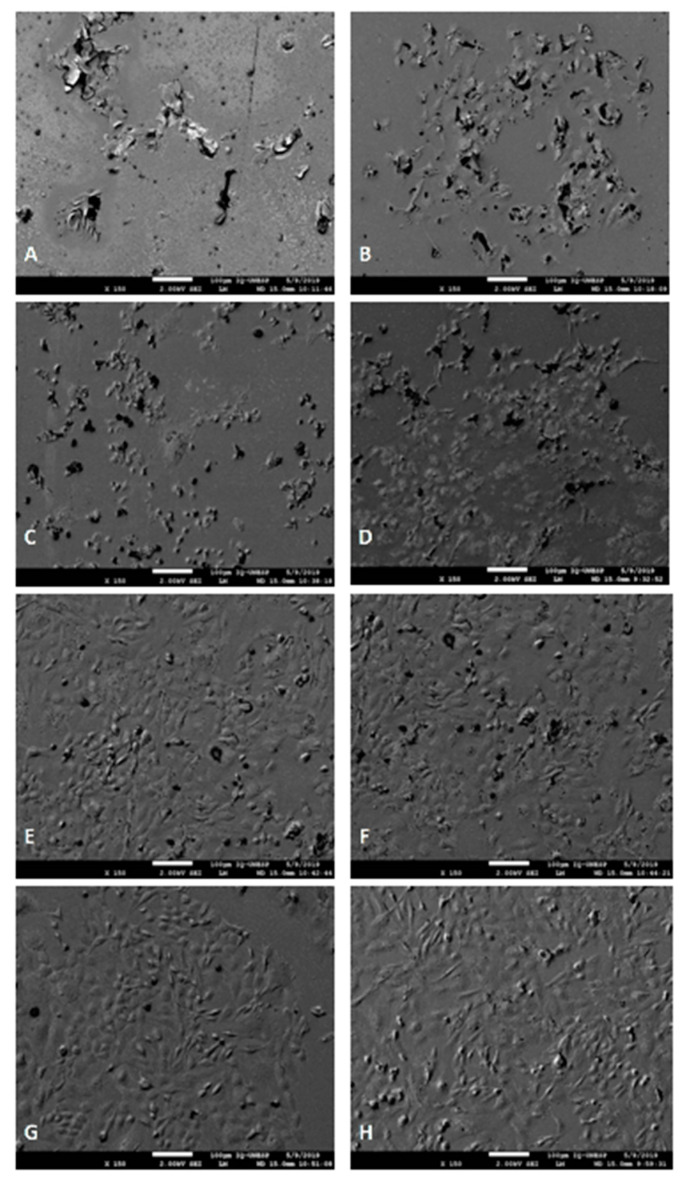
Cell morphology after 24 h and 72 h viability analysis. Legend: (**A**) Group C cell morphology after 24 h. (**B**) Cell morphology of group C after 72 h. (**C**) Cell morphology of the groups with addition of 2% HANP after 24 h. (**D**) Cell morphology of the groups with addition of 2% HANP after 72 h. (**E**) Cell morphology of the groups with addition of 5% HANP after 24 h. (**F**) Cell morphology of the groups with addition of 5% HANP after 72 h. (**G**) Cell morphology of the groups with the addition of 10% HANP after 24 h. (**H**) Cell morphology of the groups with the addition of 10% HANP after 72 h.

The evaluation of cytotoxicity was performed in two moments, described in Figure 6 and Figure 7.

There was no statistical difference between the 24 and 72 h periods. However, it can be analyzed that the higher the concentration of HANP incorporated into RMGIC, the greater the cell viability.

## 4. Discussion

RMGIC has greater mechanical resistance and better physical properties, but it has clinical limitations such as decreased biocompatibility [22,23,24] due to the presence of the 2-hydroxyethyl methacrylate (HEMA) monomer, which is considered cytotoxic in contact with the pulp tissue, thus limiting its use in deep cavities. This monomer is capable of inducing apoptosis by increasing oxidative stress induced by an excess of reactive oxygen species, damage to DNA, and suppression of cell proliferation [25].

The addition of HANP to the GIC has shown good results [10,11,21,22,25,26]. However, it is extremely important to be careful with the manipulation technique during the incorporation of HANP into the material, since improper mixing can affect its properties such as mechanical resistance [10]. Thus, the microstructural analysis of the GIC associated to NP as well as the observation of the distribution and dispersion of the same, is fundamental. From this form, three forms of manipulation of the HANP + RMGIC were defined (Table 1).

This study used SEM-FEG, which is widely used for the investigation of the surface microstructures of materials, also making possible the chemical analysis of the sample under observation [22]. In addition, this analytical method provides precision, accuracy, sensitivity, and preservation of the sample.

In all experimental groups, regardless of the technique of concentration or manipulation of the HANP, good distribution and dispersion of it was observed in the RMGIC. Due to the size of the nanomaterial, the NP can occupy small spaces, resulting in greater homogeneity of the surface and wide distribution within the material [23]. The control group and the experimental groups with the incorporation of HANP + RMGIC are shown in Figure 1.

The interaction of the chemical components of HANP + RMGIC can also be observed by x-ray dispersive energy spectroscopy, as shown in Figure 2, which shows a large amount of phosphorus (P) and calcium (Ca) in HANP (Figure 2A) compared to RMGIC alone (Figure 2B). After the incorporation of HANP into RMGIC, the chemical components were mixed (Figure 2C).

The good distribution of the NPs allows for an increase in the mechanical resistance as it promotes high density and narrowing among the particles within the ionomeric matrix [3]. Gu et al. [10] (2005) observed good distribution and dispersion of NPs within the ionomeric matrix by adding 4% or 12% HA to an encapsulated RMGIC. The authors reported the low tendency to form agglomerates, ensuring uniform distribution of NP and improvement of the mechanical properties of the material.

The porosity of the material is also an important property to be evaluated as it is related to the degree of dissolution and, consequently, to the resistance of the restorative material, which can alter its clinical durability. The increase in porosity also results in increased material roughness and greater adhesion of microorganisms [24].

In the present study, it can be seen that all groups incorporated with 10% HANP (A10, V10, and M10), regardless of the mixing mode, presented a number of pores similar to the control, so a viable explanation could be that the GIC liquid was unable to bathe all HANP as they have a larger surface area due to their nanometric size, thus making the connections insufficient to reduce the porosity of the material.

A significantly smaller number of pores was found for the other groups compared to the control (Figure 3). Groups V5 or M5 resulted in a smaller number of pores than the number observed in Group C. It is possible that the vortex and the manual technique incorporated less air compared to the technique using the amalgamator; in addition, the 5% concentration of HANP provided better chemical bonds with the GIC, resulting in less pores inside the new material. This comparison can be seen in Figure 5.

None of the groups differed from C in relation to the area occupied by the pores (Figure 4). However, a lower value was observed among the groups submitted to the vortex, indicating that, for this mixing technique, smaller pores were formed. This fact is important due to the longevity of the restorations [10,24].

The large number and area occupied by porosities can result in cracks and fractures of the material [10], and these failures can be minimized by the incorporation of 5% HANP with the help of vortex.

In addition to the physical and mechanical properties of materials, extreme importance represents cytotoxicity for the viability of a restorative material. Regardless of the incorporation technique, there was an improvement in cell viability directly proportional to the increase in the concentration of HANP (Figure 6).

Several in vitro studies have evaluated the cytotoxicity of conventional and resin-modified GICs and found that the leachable components present in the GIC were responsible for adverse effects on cell culture [8,27,28].

The isolated Vitremer showed higher cytotoxic effects compared to the groups containing HANP (Figure 6). Costa et al. (2003) [28], when evaluating five different GICs, also observed higher Vitremer cytotoxicity in MDPC-23 cells, suggesting that HEMA is the main component that contributes significantly to the cytotoxicity of this material.

Thus, the addition of HANP has shown promising results [7,10,11,15]. Pagano et al. (2019) [29] evaluated the addition of 4% HANP to a GIC and observed a significant reduction in the cytotoxicity of the material. The authors reported that this occurrence most likely occurred because HA has excellent biological behavior due to its chemical composition and crystalline structure, similar to apatite in the human skeletal system [15].

GICs are said to have low toxicity of dental pulp in clinical use. These cements exhibit cytotoxicity in the recently established state, but decrease substantially and depend on time [13], as observed in Figure 6 and Figure 7. Additionally, the addition of HANP to RMGIC may have decreased cytotoxicity due to chemical interaction, since HANP has a large amount of calcium and phosphorus (Figure 2). Noorani et al. [30], when analyzing the cytotoxicity of nanohydroxyapatite-silica, found that GIC interacted with HANP through the carboxylate groups in the polyacid [30], which can decrease the cytotoxicity of the material.

## 5. Conclusions

The HANP added to the RMGIC should be the subject of further studies as it has been shown to have good distribution and dispersion within the material. The use of vortex was the most indicated mixing method and the incorporation of 5% HANP resulted in fewer pores inside the material. Greater cell viability with the addition of higher HANP concentration could also be observed.

## Figures and Tables

**Table 1:** Groups established according to the technique of manipulation and concentration of nanohydroxyapatite and glass ionomer cement modified by resin.

Mixing Technique	HANP (%)	Groups
Control	0	C
Amalgamator (A)	2	A2
	5	A5
	10	A10
Vortex (V)	2	V2
	5	V5
	10	V10
Manual (M)	2	M2
	5	M5
	10	M10
